# Research on brownfield redevelopment based on Wuli-Shili-Renli system theory and catastrophe progression method

**DOI:** 10.1371/journal.pone.0277324

**Published:** 2022-11-17

**Authors:** He Jian, Hu Hao, Pan Haize, Liu Chuan, Li Xiaoqin, Wei Yan, Jiang Haidan, Zhang Changliang

**Affiliations:** 1 School of Civil Engineering, Chongqing University of Arts and Sciences, Chongqing, China; 2 School of Civil Engineering and Geomatics, Southwest Petroleum University, Chengdu, China; Institute for Advanced Sustainability Studies, GERMANY

## Abstract

Brownfields are an important part of urban land resources. Strengthening the governance and redevelopment of brownfields is significant to environmental protection, high-quality urban development and sustainable development. However, due to the complexity and harmfulness of brownfield pollution, the hasty, untimely, and blind development can cause serious consequences. It is infeasible to pay more attention to development than governance or vice versa. In this paper, aiming at brownfield redevelopment evaluation, we introduced the Wuli-Shili-Renli (WSR) system methodology, an oriental system thought combining qualitative and quantitative analyses, comprehensively analyzed the influencing factors of brownfields from three dimensions of Wuli, Shili and Renli, and constructed the evaluation index system of brownfield redevelopment. To avoid much subjectivity in the evaluation process, we established the evaluation model of brownfield redevelopment using the catastrophe progression method. Taking the renovation project of Shanghai Xintiandi in 1999–2001 as a reference, the evaluation index system and evaluation model were applied to the renovation project of Wenjia Street in Qingyang District, Chengdu, Sichuan Province, China in 2022, and the results provided a good basis for the decision-making process.

## Introduction

Land is the place for human existence and all activities. With the rapid development of society, the pace of industrialization and urbanization is faster and faster. People are gradually aware of the deterioration of the ecological environment while enjoying the good material conditions brought by industrialization and urbanization. In 2014, there were about 4.2 million brownfields in the EU [1]; in 2018, the United States had more than 450,000 brownfields [2]; in 2020, there were about 21,000 brownfields in England [3]. As of 2012, there were about 300,000 brownfields in China, with a total area of about 20 million hectares. With the economic development and the adjustment of urban industrial structure, the number of brownfields is increasing year by year [4]. In 2020, there were more than 7000 brownfield-related plots in Chongqing, China, covering an area of about 102 million square meters. In 2020, General Secretary Xi Jinping presided over the sixth meeting of the Central Committee for Financial and Economic Affairs and pointed out that we should promote the construction of the Chengdu-Chongqing economic circle, optimize the land spatial layout, strengthen ecological and environmental protection, and promote the high-quality development of the Chengdu-Chongqing region. However, the continuous emergence of brownfields has become the main factor affecting the urban environment and hindering the high-quality development of urban areas [5]. Generally, brownfields have superior geographical locations and great potential development values [6]. However, due to metal pollution, chemical pollution and other pollution generated by original factories in the production process [7], obstacles exist in the redevelopment and utilization, which makes it difficult to fully utilize the traffic advantages and economic benefits of brownfields. In this paper, we will focus on the scientific evaluation system of brownfield redevelopment to provide a reasonable basis for the feasibility decision of brownfield redevelopment.

## Literature review

In recent years, the social and environmental problems and potential economic benefits of brownfields have attracted increasing attention from researchers. Bieback [8] pointed out that the most effective way to solve the urban housing problem was to develop brownfields. Tiesdell [9] compared the advantages of brownfield housing development with greenfield housing development and proposed that brownfield housing development was the trend. Cao [10–12] put forward constructive suggestions on China’s brownfield issues on the basis of summarizing the experience of foreign brownfield redevelopment. In conclusion, brownfield redevelopment is an important means to solve the problem of land resources.

Currently, few methods are used in brownfield redevelopment research. Guo [13] evaluated the feasibility of brownfield redevelopment schemes using the multi-level grey evaluation method. Fang [14] and Wang [15] performed a fuzzy comprehensive evaluation to evaluate the feasibility of brownfield redevelopment schemes. Ye [16] proposed a strategic classification support system for brownfield redevelopment based on the rough set theory and multi-criteria decision theory and applied it to 201 cities in the United States; the results showed that this system could help BR project planning and budget [16]. Lesage [17] took brownfields in Montreal, Canada, as an example and proposed a modeling framework to evaluate the brownfield management decision based on the whole life cycle theory. Hou [18] used the structural equation model to prove the interests among society, economy, environment, system, stakeholders and brownfield restoration. Although these methods can evaluate the feasibility of brownfield redevelopment well, they all need the weight of each index, having strong subjectivity, complex calculation processes, a large amount of calculation and insufficient applicability. In this paper, the Wuli-Shili-Renli (WSR) system methodology was introduced into the evaluation index system of brownfield redevelopment, and the catastrophe progression method was applied to build a brownfield redevelopment evaluation model. The integration of the both formed a complete brownfield redevelopment evaluation system, and its applicability and rationality were verified in practical cases, which has good directive significance for brownfield redevelopment in China.

## Methodologies

### Wuli-Shili-Renli system methodology

As the abbreviation of “Wuli, Shili and Renli”, WSR was proposed by Professor Gu Jifa and Dr. Zhu Zhichang, famous experts in Systems Science in China, at Hull University, UK, in 1994. It is mainly used to solve complex problems [19]. The core of this methodology is to understand the objective reality of things when dealing with complex problems and use knowledge of natural science and humanities and social science to treat things well [20] to study complex problems systematically, completely and hierarchically. It is an oriental system thought combining qualitative and quantitative analysis [21].

The WSR system methodology has been introduced into various research fields [22]. Wang [23] used the WSR method to evaluate the quality of avionics software. Ji [24] used the WSR system methodology to build an intelligent building energy management model and evaluated the establishment of building energy management platforms. Luo [25] used the WSR system methodology to construct an evaluation index system of land expropriation and resettlement for social stability risk. Li [26] constructed a factor system of manufacturing energy intensity based on the WSR system method.

In the WSR system methodology, Wuli refers to the objective existence that people encounter in studying a research object, such as the environment and characteristics of the research object. Shili is the logical method used by people in studying the object, such as relevant policies and measures. Renli refers to people, the relationship among people and the behaviors of people in studying the object, such as the purposes and emotions of people and the distribution of benefits among people [27, 28]. Wuli, Shili and Renli are not independent and distinct from each other but interdependent with each other. They are the analyses of the same thing from different dimensions, which interact with and complement each other. The WSR system methodology focuses on what the system of Wuli, Shili and Renli is without deliberately paying attention to the simplicity or complexity of the problem. The basic idea of the WSR system methodology [29, 30] is shown in Table 1.

**Table 1:** The basic ideas of the WSR system methodology.

Category	Wuli	Shili	Renli
**Basic connotation**	Appearance of objective things	Mechanism of things	The relationship among people and the relationship among departments
**Theoretical basis**	Physics, laws, rules and norms	Management theory	Principles of ethics, scientific thinking and morality
**Analysis object**	The objective world, resources and constraints	Organization, system and participation mechanism	Individuals, groups, relationships, people
**Purpose**	What is it?	How to do it?	How should it be?
**Principle**	Faithful, correct and objective	Harmony, efficiency and fair competition	Humane, effective, flexible and coordinated
**Required knowledge**	Natural sciences	Management science, system science and operations research	Human and interpersonal relations, behavioral sciences and social sciences

### Applicability analysis of the WSR system methodology for brownfield redevelopment

Brownfield redevelopment involves pollution control, financial management, environmental remediation, system management, laws and regulations, etc. Due to the complex work, the project management method of brownfield redevelopment research should meet the requirements of risk assessment, sustainability and economic benefits. In this paper, we introduced the WSR system methodology into the evaluation index system of brownfield redevelopment. The WSR system methodology can comprehensively analyze the feasibility of brownfield redevelopment from Wuli, Shili and Renli to avoid neglecting key factors and effectively improve the developers’ understanding of brownfield redevelopment. In addition, it can improve the level of brownfield redevelopment management by coordinating the relationship among the three dimensions.

**Fig 1:**
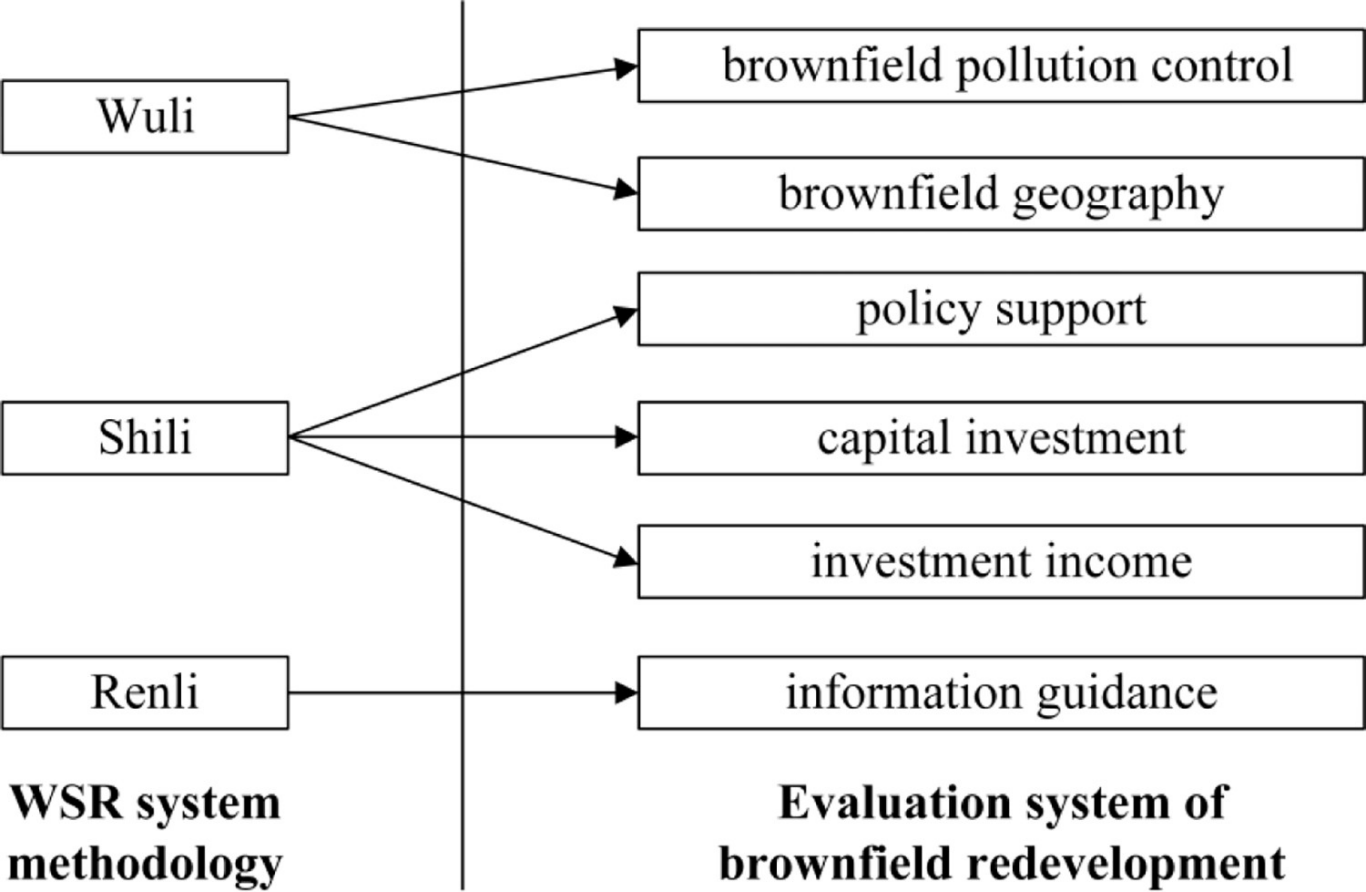
Relationship between the brownfield redevelopment evaluation system and the WSR system methodology.

Through applying the WSR system methodology, according to the three dimensions of Wuli, Shili and Renli, we analyzed the decision-making management of brownfield redevelopment from six aspects, including brownfield pollution control, brownfield geographical conditions, policy support, capital investment, investment income, and information orientation, and formulated a reasonable development plan to achieve the expected decision-making objectives. Combined with the evaluation system of the WSR system methodology (Fig 1), we redivided the evaluation index factors, enhanced the cognition of the relationship among the indexes, accurately evaluated the key evaluation indexes, and comprehensively improved the reliability of the evaluation index system of brownfield redevelopment.

### Analysis of the evaluation model of brownfield redevelopment based on the WSR system methodology

In the brownfield redevelopment evaluation system, the objective material conditions and theoretical basis in the evaluation process are supported by the Wuli dimension. Wuli is the implementation of the evaluation process, which provides a theoretical basis for Shili and a principle of communication and coordination for Renli. The Wuli dimension and the Shili dimension complement each other. The Shili dimension is combined with the objectives of the evaluation system to formulate practical and efficient policies, fund-use plans, measures and specific implementation principles, restrict the Wuli and Renli dimensions, ensure the existence form of Wuli, determine the inter-organizational relationship of the Renli dimension, and then apply effective policies, measures and principles to ensure the reliability of the evaluation system. The Renli dimension consists of various organizational relationships, interpersonal relationships and cognitive relationships; the learning of Wuli improves the cognition of brownfield redevelopment; the principle of Shili restricts the objective reality of brownfield redevelopment evaluation and ensures the operation of the evaluation system.

**Fig 2:**
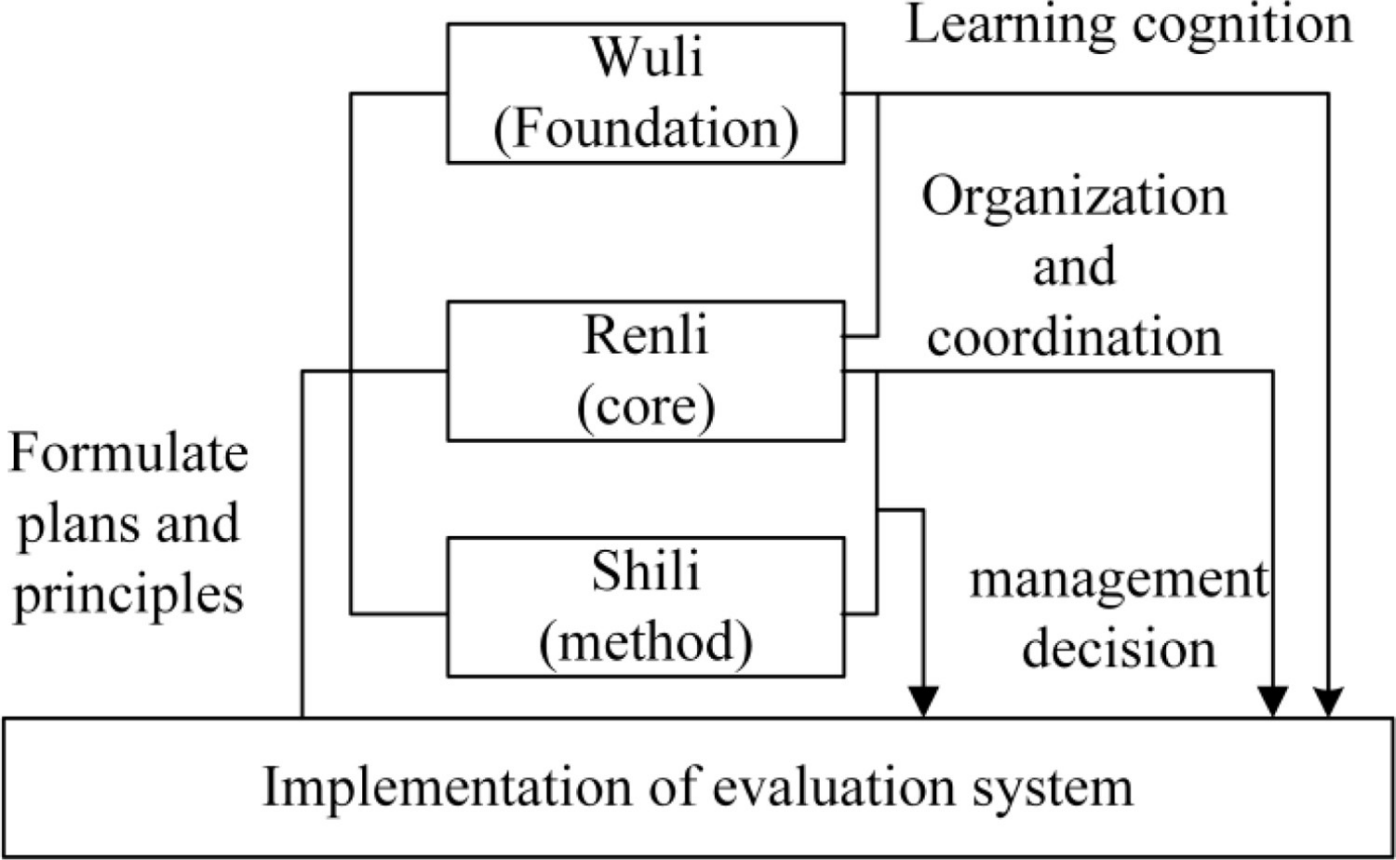
Evaluation model of brownfield redevelopment based on WSR.

In applying the WSR system methodology to evaluate brownfield redevelopment, the Wuli dimension is used as the objective learning and cognitive basis, the policies and principles of the Shili dimension as the constraint method, and the Renli dimension as the core. The three dimensions coordinate with each other and jointly promote the brownfield redevelopment evaluation to ensure the smooth evaluation process and lead to reliable evaluation results. The relationship among the three dimensions is shown in Fig 2.

### Construction of the evaluation index system of brownfield redevelopment

Through analyzing the successful cases of brownfield redevelopment, such as BP Oil Company Ruins Park, Seattle Gas Plant Park, Toronto Danglesville park, Quarry Garden in Shanghai Botanical Garden, we concluded that the key factors for the success of brownfield redevelopment are the locations, sizes and pollution of brownfields themselves, the support of governments and society, the capital investment, and the environmental protection awareness of surrounding residents. Therefore, in this paper, we combined the Wuli, Shili and Renli dimensions to establish the evaluation indexes from six aspects, including brownfield pollution control, brownfield geographical conditions, policy support, capital investment, investment income and information orientation.

### Wuli dimension

In the evaluation of brownfield redevelopment, Wuli refers to all material bases related to brownfields in brownfield redevelopment. From the perspective of brownfields themselves, the types of brownfields, pollution levels, area sizes, service conditions, geographic locations and treatment difficulties have a great impact on the brownfield government. The types of brownfields determine the difficulty of governance and the different ways of governance. According to the governance difficulty from easy to difficult, brownfields are divided into biological brownfields, chemical brownfields, physical brownfields, and radiation brownfields. From the service conditions of brownfields, more frequent use leads to more serious pollution, thus leading to greater treatment difficulty. Brownfields with less pollution can be treated preferentially to improve treatment efficiency. Brownfields with small contaminated areas are easier to manage and require fewer resources. According to the locations of the brownfields, we can judge whether the brownfield governance is convenient and feasible. If brownfields are under utilization, contain many public buildings and are frequently used for daily activities, the governance difficulty may be greatly improved. In addition to the above factors, technology is also an important factor affecting the success of brownfield management. Making reasonable plans according to local conditions is also the key to the success of brownfield management. Therefore, clarifying the information of the Wuli dimension of brownfields is the basis of brownfield redevelopment and management.

### Shili dimension

In the evaluation of brownfield redevelopment, Shili refers to the practical principles in brownfield redevelopment, including policy support, capital investment and investment income. From the successful cases of brownfield redevelopment in Britain, America and other countries, it can be seen that the governments’ attention and support are the key factors for the success of brownfield governance. The relevant legal provisions and the corresponding incentive policies issued by the governments can guarantee smooth brownfield governance. In China, although there are laws and regulations on environmental protection, there is still a lack of special laws and regulations on soil quality protection and pollution control. Therefore, only by combining the governance experience of other countries with China’s reality, issuing incentive policies, coordinating with relevant departments, and truly implementing brownfield governance, can brownfield governance be ensured.

Funds also play a decisive role in brownfield management. Funds are usually divided into government capital investment and outside investment according to the financing sources. Firstly, we should formulate the corresponding capital supervision policies to ensure the real implementation of funds. Secondly, outside investment often has many uncertain factors. Long project operation cycles, low technical contents and insufficient excavation of potential values of brownfields directly affect the credibility and investment attraction ability of projects. Sufficient attention, a sound legal system and a thorough management system are the key to attracting outside investment. In addition, the funds cannot be effectively applied in projects in the operation process, and the “secondary abandonment” caused by the wrong estimation of costs and benefits often leads to the investment of funds not meeting the governance of brownfield or excessive investment, resulting in the waste of funds. Therefore, the capital investment must be matched with brownfield governance in order to ensure effective governance.

### Renli dimension

In the evaluation of brownfield redevelopment, Renli refers to all relevant information of brownfield learned through decision-making management, organization and coordination in brownfield redevelopment, including brownfield theory cognition, brownfield harm cognition, brownfield information control, and brownfield public opinion guidance. Brownfields are a relatively new concept in China and are not known by most people. The understanding of brownfields influences the difficulty of brownfield governance and investors’ investment in brownfield redevelopment. Moreover, brownfields themselves have some characteristics. They enjoy superior geographical locations and contain or possibly contain hazardous substances. Additionally, they are difficult to redevelop or reuse. No matter from the perspective of environmental protection or development, people should be aware of the development potential of the brownfields and their harm to society. Firstly, we must understand brownfield redevelopment projects in detail and establish a mature information management system to avoid detours in the governance process. Secondly, we should actively guide public opinions on brownfield environmental protection. For the public, the information they receive directly affects their subjective views and judgments. Finally, the correct guidance of groups with certain public influence, such as authoritative institutions and certification institutions, may actively promote social support and participation.

**Fig 3:**
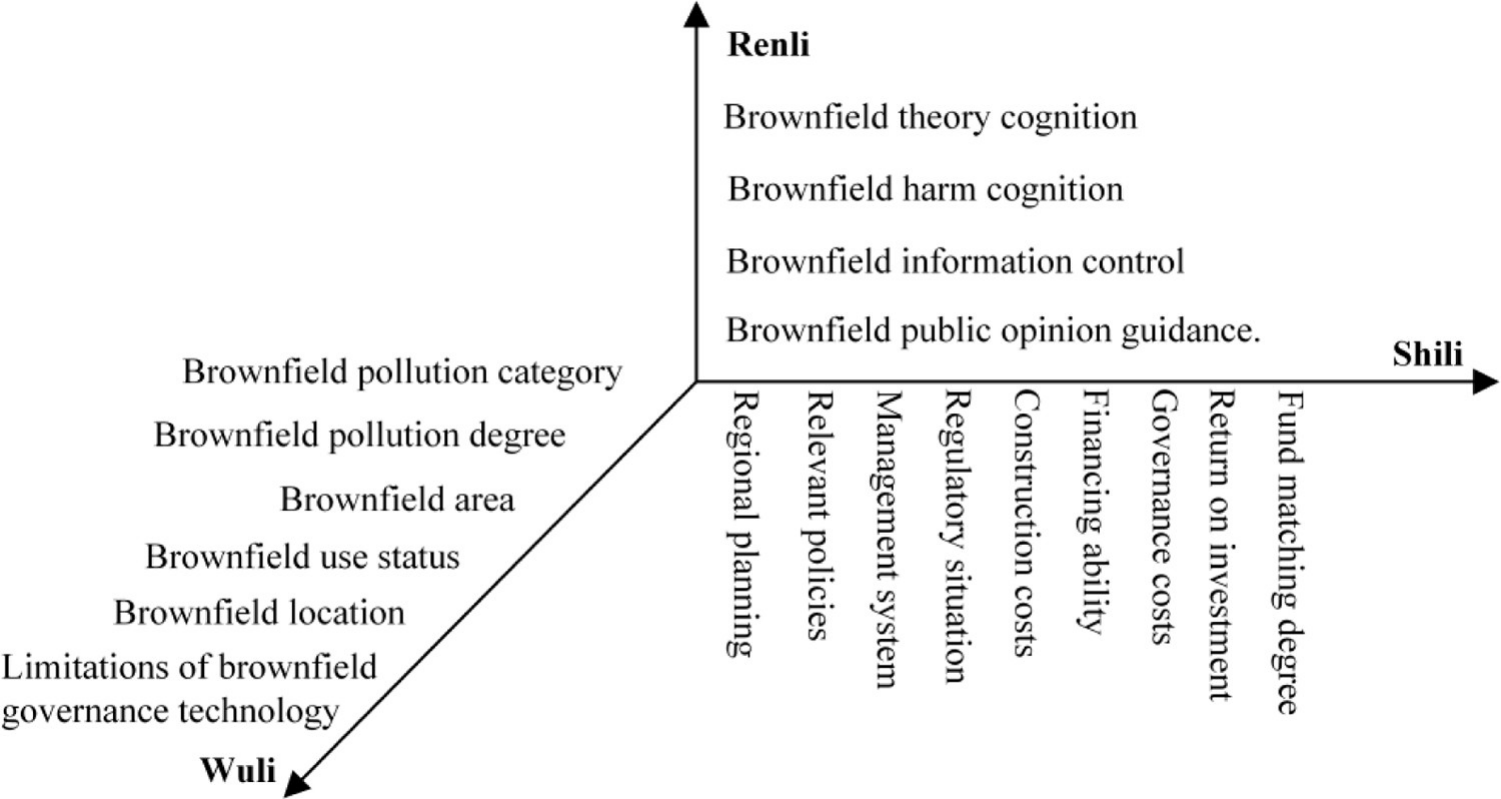
Evaluation index system of brownfield redevelopment based on the WSR system methodology.

Based on the analysis of brownfield redevelopment from the Wuli, Shili and Renli dimensions, we constructed the evaluation index system of brownfield redevelopment based on the WSR system methodology, as shown in Fig 3.

### Catastrophe theory

The founder of the catastrophe theory is Rene Thom, a French mathematician. In his book *structural stability and morphogenesis* published in 1972, he pointed out that in the process of the system from a stable state to an unstable state, with the rechange of parameters, the unstable state would enter another stable state, and the system state would change in a moment [31–33]. The catastrophe progression method is a comprehensive evaluation method developed on the basis of the elementary catastrophe theory. It combines the catastrophe theory and fuzzy mathematics to produce a catastrophe membership function, performs comprehensive quantitative operation by normalization equations, and finally reduces comprehensive quantitative operation to a parameter, that is, calculates the total membership function to complete the evaluation of the research object [34].

### Classification of catastrophe models

Catastrophe models are determined based on the evaluation index system. The types of commonly used catastrophe models include folding type, cusp type, dovetail type and butterfly type [35, 36]. The numbers of state variables and control variables of each model and the potential functions are shown in Table 2.

**Table 2:** Elementary catastrophe model.

Catastrophe type	Number of state variables	Number of control variables	Potential function
**Folding type**	1	1	$f(x)=x^{3}+ax$
**Cusp type**	1	2	$f(x)=x^{4}+ax^{2}+bx$
**Dovetail type**	1	3	$f(x)=x^{5}+ax^{3}+bx^{2}+cx$
**Butterfly type**	1	4	$f(x)=x^{6}+ax^{4}+bx^{3}+cx^{2}+dx$

### Dimensionless treatment of indexes

When the value range of each index in the evaluation system is different, the index needs to be dimensionless [37, 38]. Positive indexes mean that a larger index value is better. The dimensionless equation is:

$$y_{ij}=\frac{x_{ij}-x_{\min(j)}}{x_{\max(j)}-x_{\min(j)}}$$ (1)

Inverse indexes mean that a smaller index value is better. The dimensionless equation is:

$$y_{ij}=\frac{x_{\max(j)}-x_{ij}}{x_{\max(j)}-x_{\min(j)}}$$ (2)

where *y*_*ij*_ is the dimensionless index value, *x*_*ij*_ is the original index value, *x*_min(*j*)_ is the minimum value in the index scoring line, and *x*_max(*j*)_ is the maximum value in the index scoring line. When the index score value is within [0, 1], the indexes need no dimensionless treatment.

### Normalization of catastrophe model

Taking the cusp catastrophe model as an example, according to the catastrophe theory, when the system is in equilibrium, *f*’(*x*) = 0, that is, the equilibrium surface equation is $f^{'}(x)=4x^{3}+2ax+b=0$. The singular point set equation is obtained by calculating the second derivative of the potential function, that is, $f^{''}(x)=12x^{2}+2ax=0$. The equilibrium surface equation and the singular point set equation are simultaneously calculated, and the scalar *x* is eliminated to obtain $a=-6x^{2},b=8x^{3}$. Due to the nature of the fuzzy membership function, the value range of the control variable and the state variable must be within [0, 1]. Therefore, the equation of the cusp catastrophe model is $x_{a}=\sqrt{a},x_{b}=\sqrt[{3}]{b}$ [39, 40]. The normalization of other catastrophe models is described above, and the results are shown in Table 3.

**Table 3:** Normalization equation of catastrophe series model.

Catastrophe type	Normalization equation
**Folding type**	$x_{a}=\sqrt{a}$
**Cusp type**	$x_{a}=\sqrt{a},x_{b}=\sqrt[{3}]{b}$
**Dovetail type**	$x_{a}=\sqrt{a},x_{b}=\sqrt[{3}]{b},x_{c}=\sqrt[{4}]{\left|c\right|}$
**Butterfly type**	$x_{a}=\sqrt{a},x_{b}=\sqrt[{3}]{b},x_{c}=\sqrt[{4}]{\left|c\right|},x_{d}=\sqrt[{5}]{d}$

### Calculation of the catastrophe progression value

For the catastrophe model after normalization, the catastrophe progression value is calculated according to “complementary” or “non-complementary” principles [41, 42]. The principle of “complementarity” means that if control variables in the same system can complement each other, the average value of their function values is taken as the value of the state variable *x*; the “non-complementarity” principle means that if the control variables in the same system cannot complement each other, the minimum value is taken as the value of the state variable *x*. In order to overcome the large numerical value of the catastrophe series method, the correction relationship between the initial score value *x*_*i*_ and the comprehensive evaluation value *y*_*i*_ is shown in Table 4 [43].

**Table 4:** Correspondence between *x*_*i*_ and *y*_*i*_.

		**0.1000**	**0.2000**	**0.3000**	**0.4000**	**0.5000**	**0.5500**	**0.6000**
**Complementary**	2	0.4642	0.5848	0.6694	0.7368	0.7937	0.8193	0.8434
	3	0.5623	0.6687	0.7401	0.7953	0.8409	0.8612	0.8801
	4	0.6310	0.7248	0.7860	0.8326	0.8706	0.8873	0.9029
**Non-complementary**		0.3162	0.4472	0.5477	0.6325	0.7071	0.7416	0.7746
		**0.7000**	**0.7500**	**0.8000**	**0.8500**	**0.9000**	**0.9500**	**1.0000**
**Complementary**	2	0.8879	0.9086	0.9283	0.9473	0.9655	0.9830	1.0000
	3	0.9147	0.9306	0.9457	0.9602	0.9740	0.9873	1.0000
	4	0.9311	0.9441	0.9564	0.9680	0.9791	0.9898	1.0000
**Non-complementary**		0.8367	0.8660	0.8944	0.9220	0.9487	0.9747	1.0000

### Calculation of the evaluation index weights of brownfield redevelopment

According to the requirements of the catastrophe progression method for the evaluation index system, the indexes in the evaluation index system must be subdivided from upper indexes to lower indexes in the form of a decomposition structure. In addition, the number of the lower indexes corresponding to the upper index should not exceed four. Moreover, the indexes at the same attribute level are sorted according to the important levels. Finally, only the original values of the underlying indexes need to be obtained. In this paper, we used the AHP method to calculate the index weights.

In this article, we used the WSR system methodology to analyze the influencing factors of brownfield redevelopment from the three aspects of Wuli, Shili and Renli and followed the principle of establishing regional, predictable, quantifiable and hierarchical indexes [13] to establish an evaluation index system composed of 1 target layer, 3 primary indexes, 6 secondary indexes and 19 third-class indexes. In order to facilitate the use of the indexes, each index is simply marked, as shown in Table 5.

**Table 5:** Preliminary evaluation index system.

Target layer	Primary index	Secondary index	Third-class index
**Feasibility of brownfield redevelopment (A)**	Wuli (A_1_)	Brownfield pollution control (A_11_)	Brownfield pollution category (A_111_)
			Brownfield pollution degree (A_112_)
			Limitations of brownfield governance technology (A_113_)
		Brownfield geographical condition (A_12_)	Brownfield use status (A_121_)
			Brownfield location (A_122_)
			Brownfield area (A_123_)
	Shili (A_2_)	Policy support (A_21_)	Regional planning (A_211_)
			Relevant policies (A_212_)
			Management system (A_213_)
			Regulatory situation (A_214_)
		Capital investment (A_22_)	Construction costs (A_221_)
			Financing ability (A_222_)
			Governance costs (A_223_)
			Fund matching degree (A_224_)
		Investment income (A_23_)	Return on investment (A_231_)
	Renli (A_3_)	Information orientation (A_31_)	Brownfield theory cognition (A_311_)
			Brownfield harm cognition (A_312_)
			Brownfield information control (A_313_)
			Brownfield public opinion guidance (A_314_)

### Calculation process of the index weights

Experts from government departments, environmental protection departments, design units and construction units, university teachers and other stakeholders of brownfield redevelopment were invited to evaluate the importance of the indexes. The pairwise comparison judgment matrixes were listed according to the opinions of most experts, the weight of each index was calculated, and the consistency of the judgment matrixes was tested.

1) Calculation of the primary index weights

According to the expert evaluation, the pairwise comparison matrix of the primary indexes, Wuli (A_1_), Shili (A_2_) and Renli (A_3_), is shown in Table 6.

**Table 6:** Pairwise comparison matrix of the primary indexes.

	A_1_	A_2_	A_3_
**A**_**1**_	1	1/5	1/2
**A**_**2**_	5	1	3
**A**_**3**_	2	1/3	1

Through calculation, the weights of A_1_, A_2_, and A_3_ are 0.1220, 0.6483, and 0.2297, respectively. CI = 0.0018, and CR = 0.0032 < 0.1. The consistency test is passed, with satisfactory consistency.

2) Calculation of the secondary index weights

According to the expert evaluation, the pairwise comparison matrix of the Shili indexes policy support (A_21_), capital investment (A_22_) and investment income (A_23_) is shown in Table 7.

**Table 7:** Pairwise comparison matrix of the Shili indexes.

	A_21_	A_22_	A_23_
**A**_**21**_	1	1/5	1/7
**A**_**22**_	5	1	1/3
**A**_**23**_	7	3	1

Through calculation, the weights of A21, A22, and A23 are 0.0719, 0.2790, and 0.6491, respectively. CI = 0.0324, and CR = 0.0559 < 0.1. The consistency test is passed, with satisfactory consistency.

3) Calculation of the third-class index weights

1) Calculation of the weights of the brownfield pollution control indexes

According to the expert evaluation, the pairwise comparison matrix of the brownfield pollution control indexes brownfield pollution category (A_111_), brownfield pollution degree (A_112_), and limitations of brownfield governance technology (A_113_) is shown in Table 8.

**Table 8:** Pairwise comparison matrix of the brownfield pollution control indexes.

	A_111_	A_112_	A_113_
**A**_**111**_	1	3	1/2
**A**_**112**_	1/3	1	1/4
**A**_**113**_	2	4	1

Through calculation, the weights of A_111_, A_112_ and A_113_ are 0.3196, 0.1220 and 0.5584, respectively. CI = 0.0091, and CR = 0.0176 < 0.1. The consistency test is passed, with satisfactory consistency.

2) Calculation of the weights of the brownfield geographical condition indexes

According to the expert evaluation, the pairwise comparison matrix of the brownfield geographical condition indexes brownfield use status (A_121_), brownfield location (A_122_), and brownfield area (A_123_) is shown in Table 9.

**Table 9:** Pairwise comparison matrix of the brownfield geographical condition indexes.

	A_121_	A_122_	A_123_
**A**_**121**_	1	1/3	1/2
**A**_**122**_	3	1	3
**A**_**123**_	2	1/3	1

Through calculation, the weights of A_121_, A_122_ and A_123_ are 0.1571, 0.5936 and 0.2493, respectively. CI = 0.0268, and CR = 0.0515 < 0.1. The consistency test is passed, with satisfactory consistency.

3) Calculation of the weights of the policy support indexes

According to the expert evaluation, the pairwise comparison matrix of the policy support indexes regional planning (A_211_), relevant policies (A_212_), management system (A_213_) and regulatory situation (A_214_) is shown in Table 10.

**Table 10:** Pairwise comparison matrix of the policy support indexes.

	A_211_	A_212_	A_213_	A_214_
**A**_**211**_	1	1/3	1/5	1/7
**A**_**212**_	3	1	1/2	1/4
**A**_**213**_	5	2	1	1/2
**A**_**214**_	7	4	2	1

Through calculation, the weights of A_211_, A_212_, A_213_ and A_214_ are 0.0589, 0.1470, 0.2793 and 0.5148, respectively. CI = 0.0094, and CR = 0.0105 < 0.1. The consistency test is passed, with satisfactory consistency.

4) Calculation of the weights of the capital investment indexes

According to the expert evaluation, the pairwise comparison matrix of the capital investment indexes construction costs (A_221_), financing ability (A_222_), governance costs (A_223_) and fund matching degree (A_224_) is shown in Table 11.

**Table 11:** Pairwise comparison matrix of the capital investment indexes.

	A_221_	A_222_	A_223_	A_224_
**A**_**221**_	1	4	2	1/4
**A**_**222**_	1/4	1	1/3	1/6
**A**_**223**_	1/2	3	1	1/3
**A**_**224**_	4	6	3	1

Through calculation, the weights of A221, A222, A223 and A224 are 0.2245, 0.0638, 0.1556 and 0.5561, respectively. C = 0.0477, and CR = 0.0536 < 0.1. The consistency test is passed, with satisfactory consistency.

5) Calculation of the weights of the information orientation indexes

According to the expert evaluation, the pairwise comparison matrix of the information orientation indexes brownfield theory cognition (A_311_), brownfield harm cognition (A_312_), brownfield information control (A_313_) and brownfield public opinion guidance (A_314_) is shown in Table 12.

**Table 12:** Pairwise comparison matrix of the information orientation indexes.

	A_311_	A_312_	A_313_	A_314_
**A**_**311**_	1	1/4	1/2	1/5
**A**_**312**_	4	1	2	1/3
**A**_**313**_	3	1/2	1	1/2
**A**_**314**_	5	3	2	1

Through calculation, the weights of A311, A312, A313 and A314 are 0.0781, 0.2600, 0.1859 and 0.4760, respectively. CI = 0.0803, and CR = 0.0892 < 0.1. The consistency test is passed, with satisfactory consistency.

#### Ranking of the evaluation indexes and determination of scoring grades

Based on the above calculation results and relevant literature, each index is divided into four grades: [0.9, 0.1] (excellent), [0.8, 0.9) (good), [0.6, 0.8) (medium), and [0, 0.6) (poor). The evaluation index system and scoring standard of brownfield redevelopment are shown in Table 13.

**Table 13:** Evaluation index system and scoring standard of brownfield redevelopment.

Primary index	Secondary index	Third-class index	Score			
			[0.0, 0.6)	[0.6, 0.8)	[0.8, 0.9)	[0.90, 1.0]
**Shili (B**_**1**_ **)**	Investment income (B_11_)	Return on investment (B_111_)	Lower	Low	High	Higher
	Capital investment (B_12_)	Fund matching degree (B_121_)	Poor	Medium	Good	Excellent
		Construction costs (B_122_)	Higher	High	Low	Lower
		Governance costs (B_123_)	Higher	High	Low	Lower
		Financing ability (B_124_)	Weaker	Weak	Strong	Stronger
	Policy support (B_13_)	Regulatory situation (B_131_)	Weaker	Weak	Strong	Stronger
		Management system (B_132_)	Poor	Medium	Good	Excellent
		Relevant policies (B_133_)	Poor	Medium	Good	Excellent
		Regional planning (B_134_)	Low	Common	Secondary	High
**Renli (B**_**2**_ **)**	Information orientation (B_21_)	Brownfield public opinion guidance (B_211_)	Wrong	Partially wrong	Correct	Quite correct
		Brownfield harm cognition (B_212_)	Low	Common	Secondary	High
		Brownfield information control (B_213_)	Incomplete	Less comprehensive	More comprehensive	Quite comprehensive
		Brownfield theory cognition (B_214_)	Low	Common	Secondary	High
**Wuli (B**_**3**_ **)**	Brownfield geographical conditions (B_31_)	Brownfield location (B_311_)	Town	County	Suburb	Urban
		Brownfield area (B_312_)	< 1000 m^2^	1000 m^2^–5000 m^2^	5000 m^2^–10000 m^2^	> 10000 m^2^
		Brownfield use status (B_313_)	Abandoned	Less	Moderate	Frequent
	Brownfield pollution control (B_32_)	Limitations of brownfield governance technology (B_321_)	Harder	Difficult	Easy	Easier
		Brownfield pollution category (B_322_)	Radioactivity	Physics	Chemistry	Biology
		Brownfield pollution degree (B_323_)	More serious	Serious	Moderate	Slight

### Case analysis

**Fig 4:**
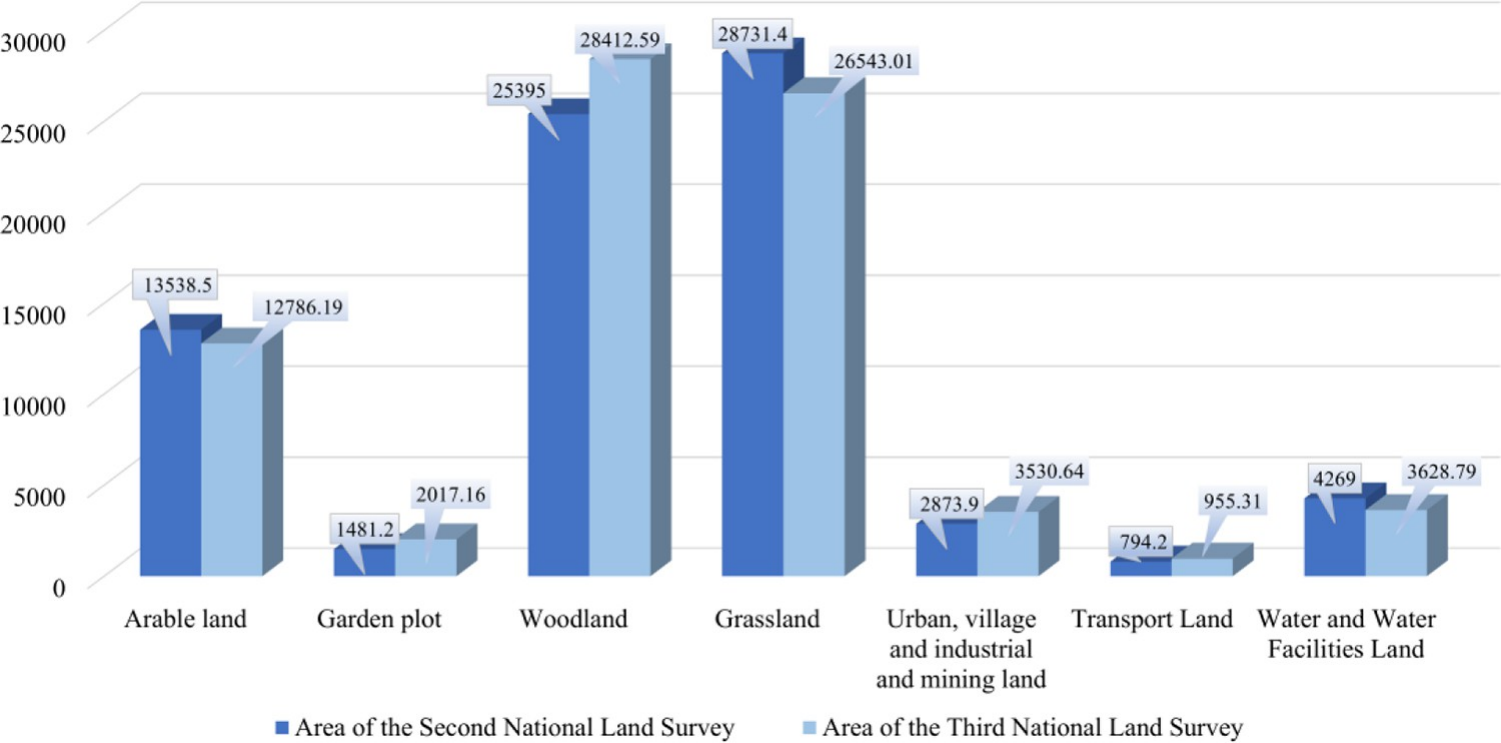
China’s second and third national land surveys (unit: million hectares).

According to the main data of China’s Third National Land Survey, as of December 31, 2019, China had 127,861,900 hm^2^ of arable land, 20,171,600 hm^2^ of garden land, 284,125,900 hm^2^ of forest land, 264,530,100 hm^2^ of grassland, 23,469,300 hm^2^ of wetland, 35,306,400 hm^2^ of urban, village and industrial and mining land, 9,553,100 hm^2^ of transportation land, 36,287,900 hm^2^ of land for water and water conservancy facilities. The data comparison between the second and third surveys on the current status of land use in China [44] is shown in Fig 4.

According to China’s Third National Land Survey, the total amount of construction land is 40,866,700 hm^2^, an increase of 8,533,300 hm^2^ or 26.5% over China’s Second National Land Survey. The GDP grew by 109.4%, and the urbanization rate of the resident population increased from 48.34% to 62.71% during this period. The increase in construction land generally coincides with the demand for land for economic and social development. However, China’s Third National Land Survey showed that the total scale of urban construction land was 10,333,300 hm^2^, indicating insufficient conservation and intensification, with a large amount of inefficient and unused land in some places and a growing trend of brownfields.

**Fig 5:**
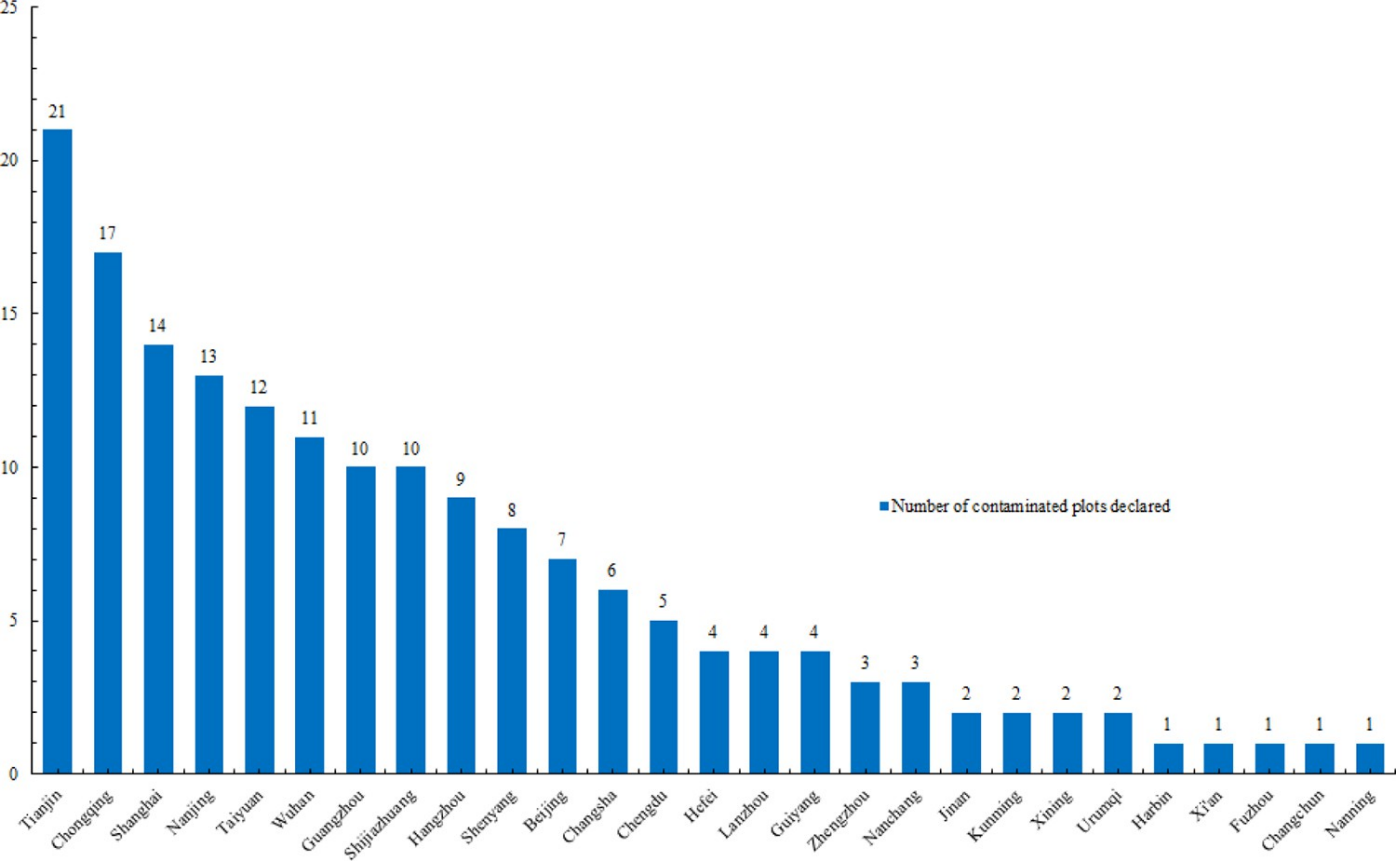
Announcement of contaminated land in major provincial capitals in China.

Since 2016, China has begun implementing a contaminated land list system to control the redevelopment of contaminated land. The *Problems and Countermeasures in the Development and Utilization of Contaminated Land in China’s Cities* shows that as of October 2018, 27 of China’s 31 provincial capitals have published risk control lists of contaminated land in their regions (**Fig 5**). According to the compilation, there are 174 contaminated plots published. Tianjin, Chongqing and Shanghai have announced the most contaminated plots, with 21, 17 and 14 plots, respectively. Due to the limited availability of land resources, many local governments have chosen to develop contaminated land with high commercial value [45].

**Fig 6:**
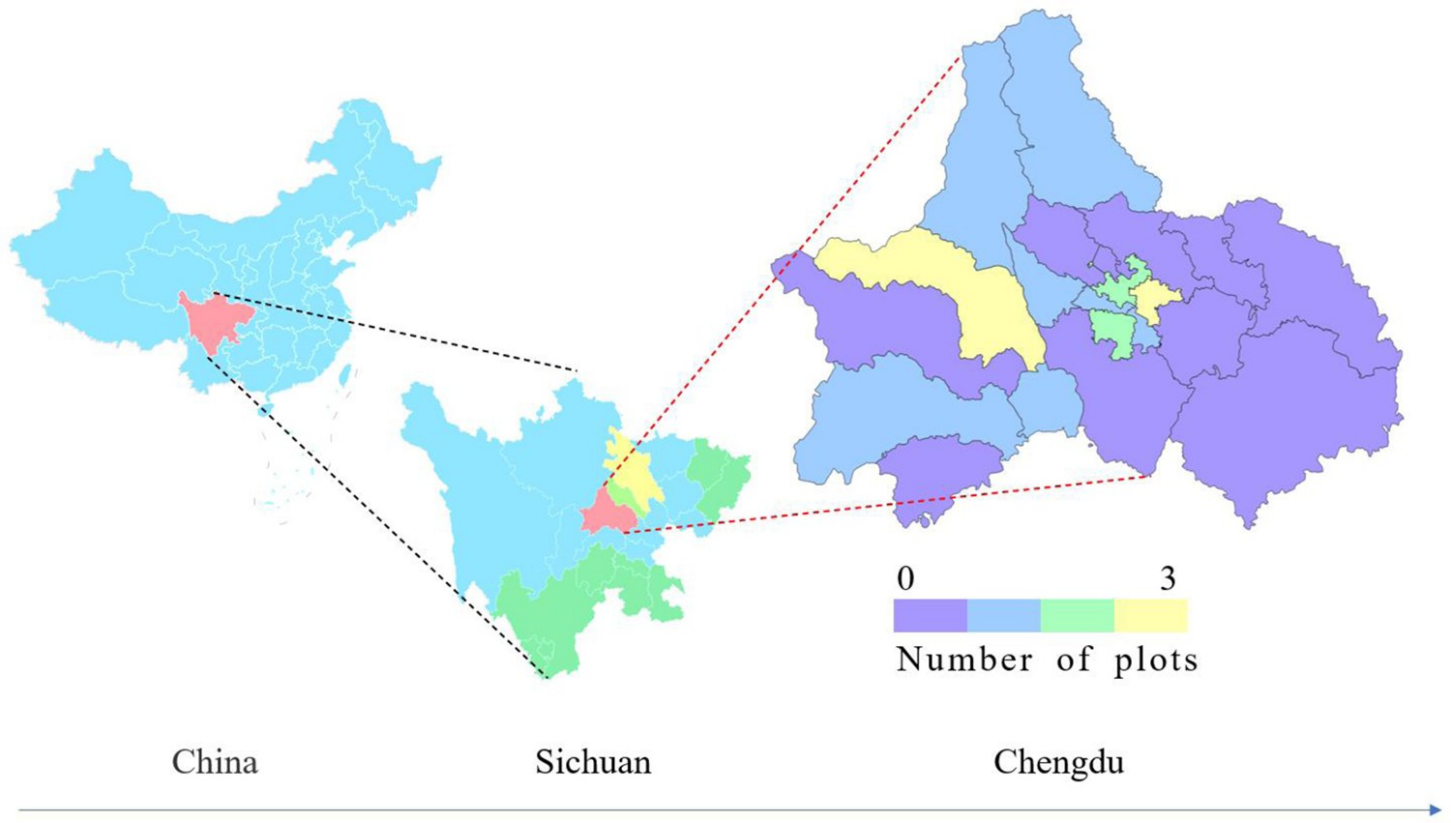
Distribution of contaminated plots in Chengdu.

Recently, the number of contaminated plots in Chengdu has continued to rise. According to the *List of the Risk Control and Remediation of Soil Contamination of Construction Land in Sichuan Province*, as of July 2022, Sichuan Province has 70 contaminated plots, and the number of contaminated plots in Chengdu has changed from 5 to 17 [46]. The distribution of contaminated plots in Chengdu is shown in **Fig 6**.

### Project overview

(1) Contrasting case—the renovation project of Shanghai Xintiandi in 1999–2001 [47]

**Fig 7:**
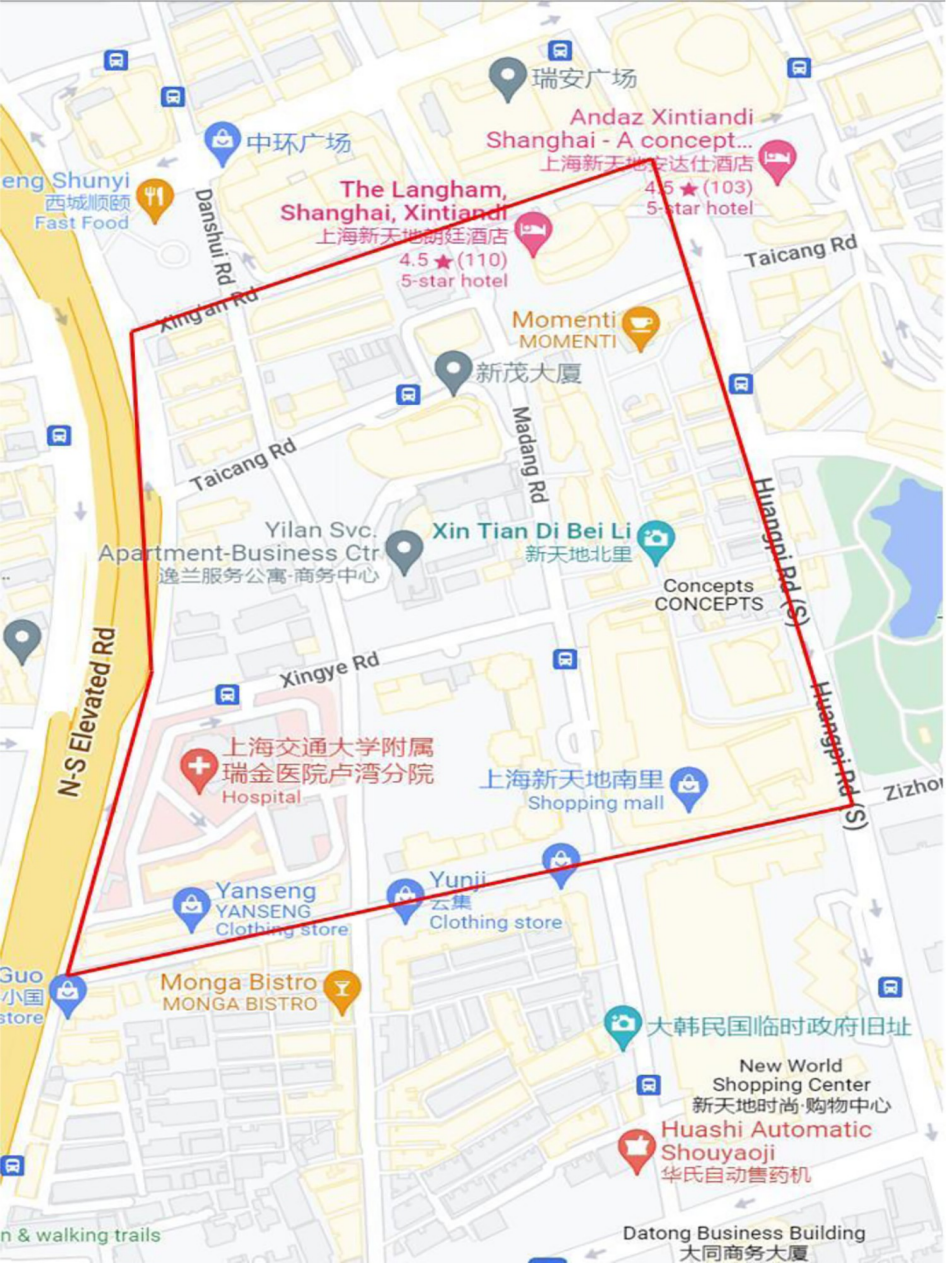
Location of the renovation project of Shanghai Xintiandi.

The earliest Xintiandi Square (**Fig 7**) is located in the Taipingqiao area, south of Huaihai Middle Road in the central district of Shanghai. It is bordered by Huangpi South Road to the east, Zizhong Road to the south, Madang Road to the west and Taicang Road to the north.

The Taipingqiao area was designated as an old city renovation area in the 1990s due to a lack of infrastructure, the disrepair of typical Shanghai Shikumen buildings, poor living environments, crowded space, and congested and isolated traffic, which made it difficult to meet the requirements of the commercial development of the central area in Shanghai at that time. The challenge was to maximize the preservation of the historical relics and culturally distinctive buildings, avoid submersion by or incompatibility with the environment after renovation, but realize the value of new commercial operations.

Based on the old Shikumen area, a landmark of modern architecture, Shanghai Xintiandi has firstly changed the original residential function of Shikumen buildings and endowed them with an innovative commercial business function, transforming these old buildings reflecting Shanghai’s history and culture into a fashionable, leisure and cultural entertainment center with an international level of dining, shopping and performing arts. In 2016, it was named by Forbes magazine as one of the top 20 civilization landmarks in the world.

(2) Study case—the renovation project of Wenjia Street in Qingyang District, Chengdu City in 2022.

**Fig 8:**
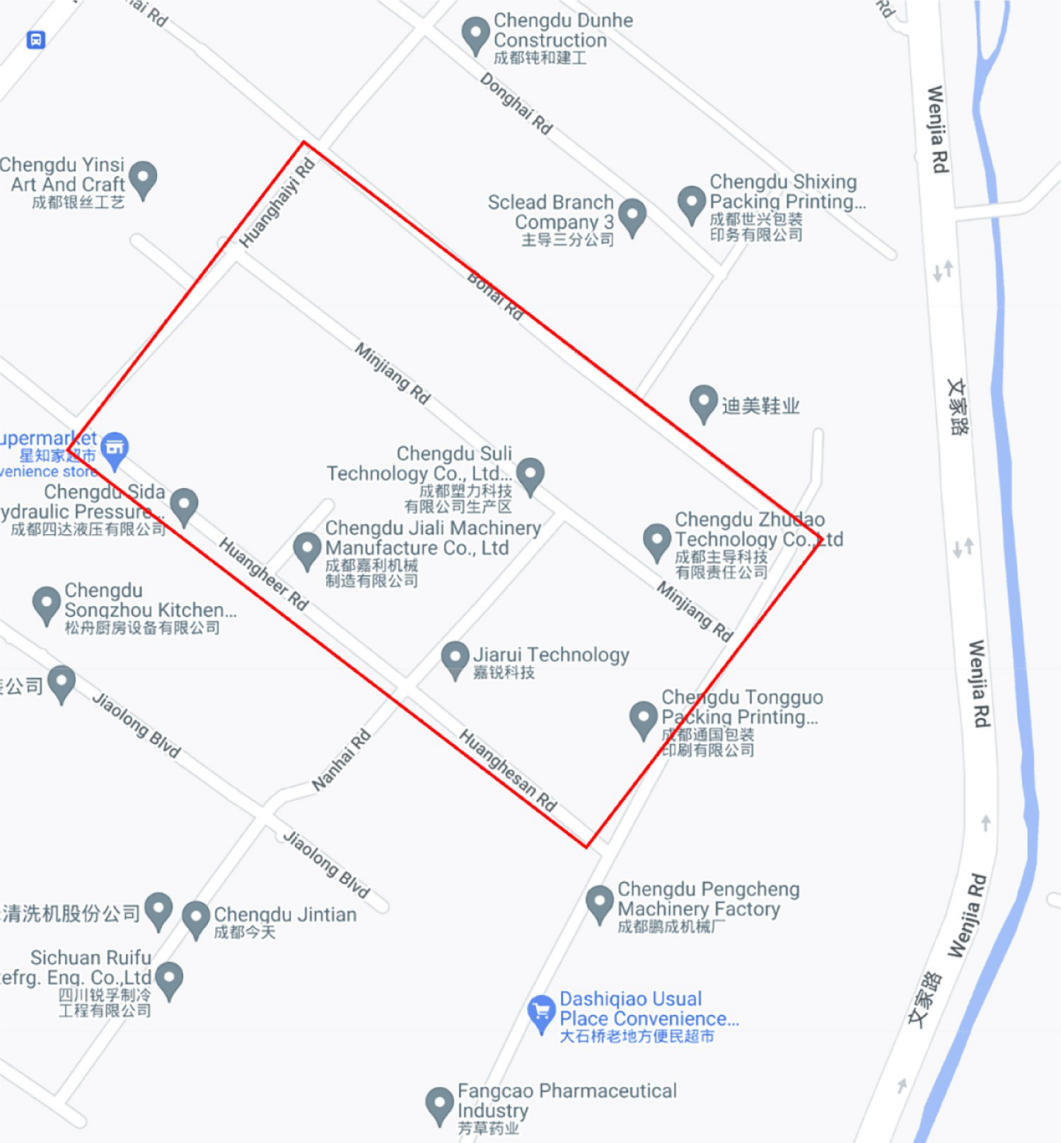
Location of the renovation project of Wenjia Street in Qingyang District, Chengdu City.

Wenjia Street (**Fig 8**) is bordered by the Supo Branch Canal and Qingyang Mould Industrial Park to the east, Yanjing Phase III Subdivision to the north, and vacant land to the west and south.

The renovation project of an area in the west of Chengdu, China, is located at the junction of the old city and the new city, with rich surrounding supporting facilities, mature medical facilities, convenient transportation, developed commerce and educational resources. It belongs to the core area of the city. The area rose in size in the 1990s. Most houses are residents’ resettlement houses, and some are small factories and workshops. Most people here are local residents. The area is characterized by dense buildings, narrow roads, small open space, dense personnel, and many pedestrians and vehicles. In recent years, with the rapid development of urban economy, the disadvantages of low-end operation, damaged and old houses, traffic congestion, ground damage, dirty and messy environment, fire hazards and chaotic rainwater drainage pipelines have gradually emerged in this area. This environment has seriously affected the image and quality of Chengdu, China. In the context of rational use of urban space and development and utilization of brownfields, the authorities have decided to renovate and upgrade this area since 2022.

Similar to the Shanghai Xintiandi project, Wenjia Street is an old neighbourhood in Chengdu, with disadvantages such as a lack of infrastructure and a poor living environment, but also features an excellent location and great development potential.

### Expert scoring

In order to evaluate the feasibility of the brownfield renovation project, various factors were evaluated according to the established evaluation index system. Six experts from the government, environmental division, construction unit, development unit, university and consulting unit were invited to score the project according to its basic characteristics. The scoring standard is based on Table 13. The final scoring result is shown in Table 14.

**Table 14:** Initial index scoring of the brownfield renovation project.

Primary index	Secondary index	Third-class index	Score						
			1	2	3	4	5	6	Mean value
**B**_**1**_	B_11_	B_111_	0.75	0.76	0.87	0.88	0.84	0.76	0.81
	B_12_	B_121_	0.82	0.60	0.78	0.74	0.60	0.83	0.73
		B_122_	0.80	0.80	0.77	0.62	0.60	0.80	0.73
		B_123_	0.70	0.80	0.80	0.80	0.82	0.80	0.79
		B_124_	0.80	0.70	0.90	0.71	0.72	0.75	0.76
	B_13_	B_131_	0.50	0.64	0.46	0.36	0.49	0.57	0.50
		B_132_	0.60	0.52	0.50	0.30	0.68	0.58	0.53
		B_133_	0.60	0.44	0.48	0.60	0.66	0.54	0.55
		B_134_	0.92	0.72	0.90	0.78	0.70	0.71	0.79
**B**_**2**_	B_21_	B_211_	0.63	0.71	0.63	0.59	0.76	0.76	0.68
		B_212_	0.50	0.36	0.40	0.28	0.37	0.54	0.41
		B_213_	0.60	0.60	0.72	0.58	0.65	0.62	0.63
		B_214_	0.50	0.23	0.41	0.27	0.26	0.27	0.32
**B**_**3**_	B_31_	B_311_	0.92	0.94	0.98	0.95	0.99	1.00	0.96
		B_312_	0.83	0.79	0.90	0.89	0.71	0.72	0.81
		B_313_	0.84	0.85	0.85	0.73	0.77	0.74	0.80
	B_32_	B_321_	0.80	0.85	0.84	0.83	0.81	0.66	0.80
		B_322_	0.70	0.69	0.60	0.62	0.72	0.63	0.66
		B_323_	0.82	0.80	0.87	0.77	0.90	0.79	0.83

### Calculation of the value of the catastrophe membership function

In this paper, the index value is within [0, 1], so dimensionless treatment is not required. According to the catastrophe model shown in Table 2, the normalization equation shown in Tables 2, 3 and the “complementary” and “non-complementary” principles, the catastrophe membership function values of the secondary indexes, primary indexes and target layer were obtained.

(1) Calculation of the secondary indexes

Capital investment B_12_ consists of four indexes and is of the butterfly type. The indexes complement each other, then:

$$B_{12}=(\sqrt{B_{121}}+\sqrt[{3}]{B_{122}}+\sqrt[{4}]{B_{123}}+\sqrt[{5}]{B_{124}})/4=0.9110$$

Policy support B_13_ is composed of four indexes and is of the butterfly type. There is complementarity among the indexes, then:

$$B_{13}=(\sqrt{B_{131}}+\sqrt[{3}]{B_{132}}+\sqrt[{4}]{B_{133}}+\sqrt[{5}]{B_{134}})/4=0.8329$$

Information orientation B_21_ consists of four indexes and is of the butterfly type. The indexes complement each other, then:

$$B_{21}=(\sqrt{B_{211}}+\sqrt[{3}]{B_{212}}+\sqrt[{4}]{B_{213}}+\sqrt[{5}]{B_{214}})/4=0.8137$$

Brownfield geographical condition B_31_ is composed of three indexes and is of the dovetail type. There is no complementarity among the indexes, then:

$$B_{31}=\min(\sqrt{B_{311}},\sqrt[{3}]{B_{312}},\sqrt[{4}]{B_{313}})=0.8944$$

Brownfield pollution control B_32_ consists of three indexes and is of the dovetail type. The indexes do not complement each other, then:

$$B_{32}=\min(\sqrt{B_{321}},\sqrt[{3}]{B_{322}},\sqrt[{4}]{B_{323}})=0.8124$$

(2) Calculation of the primary indexes

Shili B_1_ is composed of three indexes and is of the dovetail type. There is complementarity among the indexes, then:

$$B_{1}=(\sqrt{B_{11}}+\sqrt[{3}]{B_{12}}+\sqrt[{4}]{B_{13}})/3=0.9416$$

Wuli B_3_ consists of two indexes and is of the cusp type. The indexes complement each other, then:

$$B_{3}=\min(\sqrt{B_{31}},\sqrt[{3}]{B_{32}})=0.9013$$

(3) Calculation of the comprehensive evaluation index

Comprehensive evaluation index B is composed of three indexes and is of the dovetail type. The indexes complement each other, then:

$$B_{}=(\sqrt{B_{1}}+\sqrt[{3}]{B_{2}}+\sqrt[{4}]{B_{3}})/3=0.9594$$

Comparing the results of the comprehensive evaluation index, primary indexes and secondary indexes with Table 4, we obtained the final evaluation results of the feasibility of the brownfield renovation project, as shown in Table 15.

**Table 15:** Evaluation results of the feasibility of the brownfield redevelopment project.

		*y*	*x*	Grade
**Comprehensive evaluation index**	B	0.9594	0.8472	Ⅱ
**Primary index**	B_1_	0.9416	0.7861	Ⅲ
	B_2_	0.8137	0.8137	Ⅱ
	B_3_	0.9013	0.7322	Ⅲ
**Secondary index**	B_11_	0.8100	0.8100	Ⅱ
	B_12_	0.9110	0.6275	Ⅲ
	B_13_	0.8329	0.4008	Ⅳ
	B_21_	0.8137	0.3567	Ⅳ
	B_31_	0.8944	0.8000	Ⅱ
	B_32_	0.8124	0.6600	Ⅲ

## Results and discussion

According to the evaluation results in Table 15, the redevelopment feasibility grade of the brownfield is good, indicating that the brownfield is suitable for redevelopment. It can be seen from the index scores of each level that the Renli evaluation level is good in the three aspects, indicating that the concept of environmental protection has been strengthened, the requirements for the living environment are getting higher, and the desire for community transformation is stronger. It can be seen from the scores of each secondary index that among the six aspects of brownfield pollution control, brownfield geographical conditions, policy support, capital investment, investment income and information orientation, the evaluation score of the investment income is the highest, and the investment income brought by the redevelopment of the project is the most obvious. In the capital investment, the matching degree of investment and brownfield governance and the implementation of relevant measures of local government investment are also obvious advantages. The second is the brownfield geographical conditions, and the third is the brownfield pollution control, showing that the location advantage of the brownfield is significant, the pollution of the brownfield can be well dealt with, and the overall conditions of the brownfield are superior. Generally speaking, there is still a lack of policy support. Although attention is paid to the overall regional planning, the management system, supervision and implementation ability and supporting policies for the brownfield renovation project need to be strengthened. From the perspective of information orientation, the overall score of the brownfield renovation project is not high, indicating that the public’s understanding of brownfield knowledge and the harm of brownfield pollution need to be improved.

## Conclusions and future research orientations

In this paper, we regarded the brownfield redevelopment evaluation index system as an interrelated organic whole and constructed the brownfield redevelopment evaluation model by combining the WSR system methodology with the catastrophe progression method.The feasibility of the brownfield redevelopment evaluation system established in this paper was verified by an actual case. In the process of brownfield redevelopment research, the following conclusions were drawn:

1. The WSR system methodology was applied to the feasibility evaluation of brownfield redevelopment, and the problem of brownfield redevelopment was analyzed from the three dimensions of Wuli, Shili and Renli. Not only the conditions and characteristics of the brownfield were considered, but also the policies, regulations and relevant information affecting the decision-making of brownfield redevelopment were considered. Moreover, the coordination of the three dimensions enhanced the reliability of the evaluation index system of brownfield redevelopment.
2. The catastrophe progression method was applied to the feasibility evaluation of brownfield redevelopment. Considering the interaction among the indexes, we calculated the index values of the upper indexes layer by layer through the score values of the lower indexes, obtained the importance ranking of the indexes, effectively reducing the subjective error caused by the calculation of index weights, and had a more objective evaluation of all aspects of brownfield redevelopment. The developers can comprehensively consider the response measures of various influencing factors in the whole process of brownfield redevelopment.
3. Brownfield redevelopment involves many stakeholders, and the conditions of brownfields and the surrounding environments are complex. Therefore, future studies on brownfield redevelopment should focus on how to reasonably divide the rights and responsibilities of stakeholders and scientifically formulate policies and legal measures to effectively solve their conflict of interests; in addition, during development evaluation, establishing a more detailed and data-based evaluation index system and forming an information system will be the key to the next research on brownfield redevelopment.
