# Correction: Research on brownfield redevelopment based on Wuli-Shili-Renli system theory and catastrophe progression method

**DOI:** 10.1371/journal.pone.0308126

**Published:** 2024-07-25

**Authors:** He Jian, Hu Hao, Pan Haize, Liu Chuan, Li Xiaoqin, Wei Yan, Jiang Haidan, Zhang Changliang

The published funding statement is incorrect. The correct funding statement is as follows: This study was supported by the Science and Technology Research Project of Chongqing Municipal Education Commission (Grant Nos. KJQN202101337 and KJQN202101322), the School-level Scientific Research Project of Chongqing University of Arts and Sciences (No. 2017YJ354), the Natural Science Foundation of Chongqing (No. cstc2019jcyj-msxmX0440), and the Major Breeding Project of Chongqing University of Arts and Sciences (No. P2018JG13).
